# Diagnosis, treatment, and follow-up of patients with cerebral amyloid angiopathy-related inflammation

**DOI:** 10.1007/s10072-022-06299-y

**Published:** 2022-08-05

**Authors:** Virginia Cancelloni, Alessandra Rufa, Carla Battisti, Nicola De Stefano, Egidio Mastrocinque, Guido Garosi, Duccio Venezia, Ivano Chiarotti, Alfonso Cerase

**Affiliations:** 1 Unit of Neurology and Neurometabolic Disorders, Clinical Department of Neurological and Motor Sciences & University Department of Medicine, Surgery and Neuroscience, Azienda ospedaliero-universitaria Senese & University of Siena, “Santa Maria alle Scotte” NHS & University Hospital, 53100 Siena, Tuscany Italy; 2 Unit of Anesthesiology, Emergency-Urgency Intensive Care and Transplants, Clinical Department of Emergency-Urgency and Transplants, Azienda Ospedaliero-universitaria Senese, “Santa Maria alle Scotte” NHS & University Hospital, 53100 Siena, Tuscany Italy; 3 Unit of Nephrology, Dialysis, and Transplants, Clinical Department of Emergency-Urgency and Transplants, Azienda ospedaliero-universitaria Senese, “Santa Maria alle Scotte” NHS & University Hospital, 53100 Siena, Tuscany Italy; 4 Unit of Radiology, Department of Diagnostic Imaging, Azienda USL Toscana sud est, “San Donato” NHS Hospital, 52100 Arezzo, Tuscany Italy; 5 Unit of Neuroimaging – Diagnostic and Functional Neuroradiology, Clinical Department of Neurological and Motor Sciences, Azienda ospedaliero-universitaria Senese, “Santa Maria alle Scotte” NHS & University Hospital, 53100 Siena, Tuscany Italy

**Keywords:** Amyloid-related imaging abnormalities, Cerebral amyloid angiopathy-related inflammation, Computed tomography, Magnetic resonance imaging, Spontaneous remission, Treatment

## Abstract

**Purpose:**

Cerebral amyloid angiopathy-related inflammation (CAA-ri) is a rare potentially reversible encephalopathy associated with an autoimmune process against proteins deposited in the walls of cortical and leptomeningeal brain vessels. Definite diagnosis requires histopathological features of vascular inflammation and amyloid deposition from brain biopsy. Clinical-neuroradiological criteria have been recently introduced and validated to reduce the need for biopsy. The purpose of this paper is to report a historical retrospective review of clinical-neuroradiological follow-up of two patients with probable CAA-ri and five patients with a reasonably probable suspect of CAA-ri (4 females, 3 males, patient’s age at admission: 66–79 years) seen at our institution between 2007 and 2021, focusing on clinical and neuroradiological awareness to this entity and variable response to immunotherapy.

**Materials and methods:**

Clinical features at presentation included subacute to acute confusion (6/7), seizures (4/7), cognitive impairment (5/7), and focal neurological signs (3/7). Neuroradiology included braincomputed tomography followed by magnetic resonance imaging. Infectious diseases and autoimmune workups were then performed.

**Results:**

CSF analysis was performed in two patients. Cerebral angiography was performed in two patients, to rule out vascular malformations. Hemorrhagic posterior reversible encephalopathy syndrome has been suspected in two patients. Four patients underwent immunotherapy with corticosteroids followed by reduction of brain dysfunctions. Three patients did not undergo immunotherapy but underwent clinical and/or neuroradiological remission.

**Conclusions:**

Patients with CAA-ri present a rare steroid-responsive acute to subacute brain dysfunction. Thus, it has to be known and recognized both clinically and neuroradiologically. Spontaneous clinical and/or neuroradiological improvement is possible in patients with mild symptoms.

## Introduction

Cerebral amyloid angiopathy is a common disorder of the elderly in which amyloid peptides settle on cerebral vessels with consequent vascular fragility and destruction [1], leading to cerebral hemorrhage as the most common presentation. Coexisting inflammation including vasculitis or perivasculitis and cerebral edema has been described firstly in 1974 [2] and then called CAA-related inflammation (CAA-ri) [3–6]. Etiopathogenesis of CAA-ri still remains unknown, although the similarities to amyloid-related imaging abnormalities (ARIA) [7] observed in some participants within immunization clinical trials for Alzheimer disease support an autoimmune mechanism of inflammation [7–9]. Clinically, CAA-ri typically presents in patients aged ≥ 40 years, with acute/subacute onset of ≥ 1 of the following clinical features: headache, decrease in consciousness, behavioral changes, cognitive decline, seizures or focal neurological signs, and not directly attributable to an acute intracerebral hemorrhage [3]. According to current criteria [6], the diagnosis of probable CAA-ri strictly requires clinical [6], neuroradiological [10], and biochemical [6] findings including (i) magnetic resonance imaging (MRI) T2*-weighted gradient-echo (GE) images or susceptibility-weighted imaging (SWI) showing at least one imaging marker such as cerebral microbleeds (CMBs) or cortical superficial siderosis (CSS), (ii) MRI fast fluid-attenuated inversion recovery (FLAIR) images showing corticosubcortical monolateral or bilateral hyperintensities suggestive of vasogenic edema or sulcal effusion, (iii) clinical features associated with MRI findings, and (iiii) cerebral spinal fluid (CSF) testing to rule out infections or tumors. Notably, neoplastic, infectious, or other causes have to be ruled out. Immunotherapy results in dramatic clinical and neuroradiological improvement; however some patients minimally symptomatic or with spontaneous remission have been described [7].

The purpose of this paper is to report about two patients with probable CAA-ri and five patients with a probable suspect of CAA-ri, focusing on the difficulty of diagnosis and treatment options.

### Patients

The seven patients have been seen in our institution between 2007 and 2021 and have been identified by the search of terms such as “cortical microbleeds” and “vasogenic edema,” eventually associated with “amyloidosis,” “inflammation,” “amyloidosis-related inflammation”, “inflammatory amyloidosis,” or “inflammation related to amyloidosis” in a single neuroradiological report present in the Radiological Information System of our institution and then matched with clinical features and laboratory findings.

All the patients underwent unenhanced and/or iodine contrast-enhanced brain-computed tomography (CT); CT angiography has been performed in 2/7 patients. 1.5T brain MRI was then performed in all the patients during hospitalization. At diagnosis, the patients underwent FLAIR (7/7), SWI (6/7), and GE (2/7) images of the brain. Digital subtraction angiography (DSA) was performed in two patients. All the patients underwent blood examination including hemochrome, inflammatory indexes, liver and renal function, muscular enzymes, coagulation, electrolytes, cholesterol, and thyroid function; biochemical tests for principal neurotropic agents and autoimmune encephalitis were performed in four and two patients, respectively. Lumbar puncture for CSF analysis was performed in two patients. At follow-up, four patients underwent MRI and one CT; the remaining two patients did not undergo neuroradiological follow-up.

**Fig. 1:**
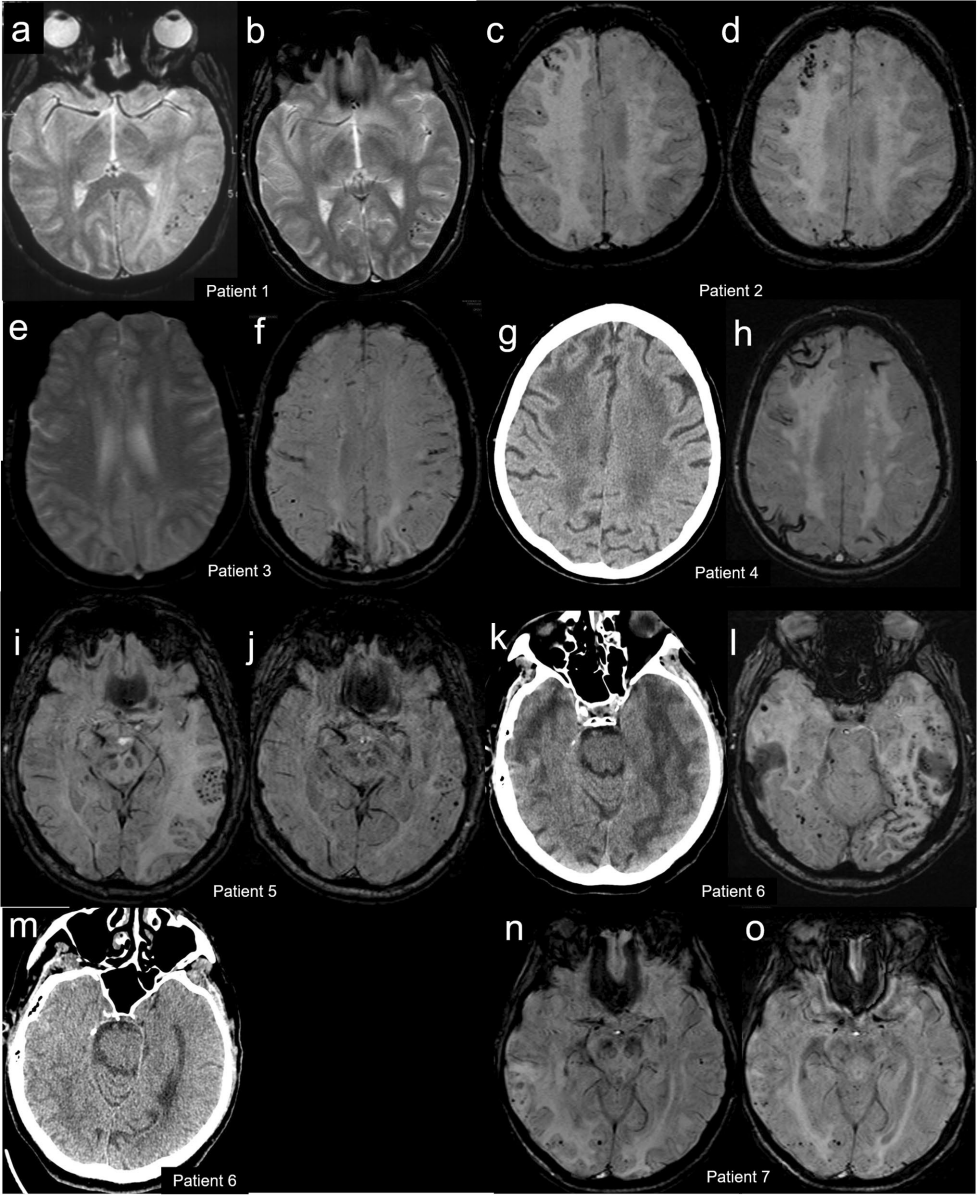
Neuroradiological findings. Patient 1: gradient echo magnetic resonance (MR) axial images at diagnosis (**a**) and 1.5-month follow-up (**b**). Patient 2: susceptibility-weighted MR axial images at diagnosis (**c**) and 1-month follow-up (**d**). Patient 3: gradient echo (**e**) and susceptibility-weighted (**f**) MR axial images at diagnosis. Patient 4: computed tomography (**g**) and susceptibility-weighted MR (**h**) axial images at diagnosis. Patient 5: susceptibility-weighted MR axial images at diagnosis (**i**) and 3-month follow-up (**j**). Patient 6: computed tomography (**k**) and susceptibility-weighted MR (**l**) axial images at diagnosis and computed tomography axial image at 5-month follow-up (**m**). Patient 7: susceptibility-weighted MR axial images at diagnosis (**n**) and 1-month follow-up (**o**)

Patient 1 presented in February 2007. She was a 66-year-old woman with a medical history of mammalian cancer and rheumatic polymyalgia. She presented to the Emergency Room of another institution for subacute confusion, memory complaints, aphasia, and afebrile generalized tonic–clonic seizure. Brain CT showed vasogenic edema in the left temporo-occipital area. She was admitted to our institution for cerebral digital subtraction angiography (DSA) which was negative for both vascular malformation and large- to medium-sized vessel vasculitis. Brain MRI showed that the left temporo-occipital vasogenic edema was associated with CMBs (Fig. 1a). Blood examination was normal. Lumbar puncture was not performed. Revision of clinical and MRI findings led to consider reasonable a probable suspect of CAA-ri; she was treated with intravenous dexamethasone (4 mg/day for 3 days). She returned almost asymptomatic. 1.5-month brain MRI follow-up (Fig. 1b) showed clearcut reduction of vasogenic edema.

Patient 2 presented in February 2012. She was a 79-year-old woman with a history of hypertension and dyslipidemia with a 6-month history of afebrile-generalized seizures, left hemisome hypoesthesia, and progressive cognitive impairment. She underwent outpatient brain MRI which revealed multiple bilateral frontal, parietal, and occipital CMBs at SWI (Fig. 1b), associated with right temporo-occipital white matter vasogenic edema at FLAIR. These abnormalities were initially suspected as vasogenic edema secondary to a dural arteriovenous fistula or cerebral vasculitis. Blood analyses were normal. She was admitted to our department for brain CT and cerebral DSA which was negative for vascular malformation or large- to medium-sized vessel vasculitis. Lumbar puncture was not performed. Revision of clinical and MRI findings led to consider reasonable a probable suspect of them consistent with probable CAA-ri. Immunotherapy with dexamethasone (8 mg/d for 15 days followed by a slow taper) was administered. Two-month brain MRI follow-up (Fig. 1c) showed a significant reduction of parenchymal abnormalities with persisting microbleeds.

Patient 3 presented in September 2013. She was a 69-year-old woman with a history of slight memory complaints, previous cerebral hemorrhage, and afebrile generalized tonic–clonic seizures. She was admitted to the Emergency Room of our institution for subacute confusion, apraxia, spatiotemporal disorientation, ataxia, and visual complaints. Blood was abnormal for ESR (41 mm/h; normal 0–35), fibrinogen (566 mg/dl; normal 150–469), and CPK (168 UI/l; normal 10–140). Electroencephalogram (EEG) pointed out right temporal-parietal-occipital focal pseudoperiodical activity. Following brain CT, brain MRI revealed right frontal-parietal-occipital and left temporal-parietal-occipital vasogenic edema, bilateral frontoparietal (Fig. 1e,f), temporo-occipital, and cerebellar CSS, leading to consider reasonable a probable suspect of consistent with probable CAA-ri. Lumbar puncture was not performed. Neither immunotherapy nor specific antiepileptic medications other than home therapy were administered. During the recovery, clinical condition, and in particular confusion and disorientation, gradually improved. One-week EEG follow-up pointed out a significant reduction of pseudoperiodical activity. A follow-up brain MRI could not be performed due to the patient’s non-collaboration.

Patient 4 presented in July 2019. She was a 75-year-old woman with a history of progressive cognitive decline and dyslipidemia. She was admitted for acute and transient confusion and spatiotemporal disorientation. Blood analyses were abnormal for CRP (1.17 mg/dl; normal 0–0.5) and hemoglobin (11.8 g/dl; normal 12.8–18). Serological evaluation for HIV, *Borrelia burgdorferi*, and *Treponema pallidum* was negative. The serum genotype of apolipoprotein E (APOE) was ε2/ε3. Brain CT (Fig. 1g) showed diffuse chronic leukoencephalopathy and vasogenic edema and thickening of the right frontal lobe. Brain MRI (Fig. 1h) added signs of CMBs and CSS at SWI, leading to consider reasonable a probable suspect of CAA-ri. Lumbar puncture was not performed. She underwent significant spontaneous recovery without immunotherapy. Brain MRI follow-up could not be performed due to the patient’s non-collaboration.

Patient 5 presented in August 2019. He was a 69-year-old man with a history of hypertension. He presented to the Emergency Room of our institution for acute confusion, right hemiparesis, and generalized tonic–clonic seizures. Because of a progressive loss of consciousness, he was intubated and admitted to the Intensive Care Unit. Following brain CT, brain MRI revealed subcortical left temporal-parietal-occipital and right temporoparietal vasogenic edema and multiple bilateral CMBs more evident in the left temporal area (Fig. 1j). These abnormalities were initially interpreted as hemorrhagic posterior reversible encephalopathy syndrome (PRES). Blood examination was negative for principal neurotropic agents and autoimmune encephalitis. CSF analysis showed raised protein (81.80 mg/dl; normal 20–40) and glucose (112 mg/dl; normal 40–70) but normal cell count in the CSF. CRP for principal neurotropic agents was negative. Revision of clinical features and brain MRI findings led to the diagnosis of probable CAA-ri. Then, he was treated with intravenous methylprednisolone 1 g/d for 5 days followed by oral prednisone 80 mg/d with slow dose reduction. Confusion and hemiparesis gradually resolved. One month later, a follow-up brain MRI (Fig. 1k) showed an impressive reduction of vasogenic edema. At the same time, the patient was asymptomatic except for mild right hemiparesis.

Patient 6 presented in November 2019. He was a 70-year-old man with a history of depression, iatrogenic parkinsonism, and severe chronic renal failure leading to kidney transplantation requiring immunosuppressive therapy with cyclosporine and mycophenolate. He presented to the Emergency Room of our institution for subacute mild transient confusion and aphasia. Blood was abnormal for creatinine (1.5 mg/dl, normal 0.7–1.2), and hemoglobin (11.6 g/dl; normal 12.8–18). Serum CRP for JC virus and CMV was negative. Brain CT showed large areas of vasogenic edema in both the cerebral hemispheres (Fig. 1k). Brain MRI showed bilateral temporal-parietal-occipital vasogenic edema and white matter hyperintensities, CMBs and CSS mainly in the left temporoparietal area (Fig. 1l). Lumbar puncture was not performed. Due to the patient’s medical history, the first suspicion was hemorrhagic PRES, but a careful revision of clinical and MRI findings led to consider reasonable a probable suspect of CAA-ri. No immunotherapy was administered. Clinical condition gradually improved. Five-month follow-up CT showed a clear-cut reduction of cerebral edema (Fig. 1m). Then, a cholangiocarcinoma appeared and led the patient to death 1 year later.

Patient 7 presented in August 2021. He was a 72-year-old man with a history of essential thrombocythemia and prostatic cancer treated by surgery. He was admitted for acute confusion. Neurologic examination revealed a soporous state, spatiotemporal disorientation, left hemiparesis, pyramidal signs in the lower limbs, and neglect. Brain CT showed bilateral and extensive subcortical fronto-temporo-parietal hypodensity, more evident in the right cerebral hemisphere. Brain MRI confirmed subcortical bilateral frontal–temporal-parietal FLAIR white matter hyperintensity, with cortical sparing. SWI (Fig. 1n) showed multiple bilateral CMBs more evident in the affected areas, consistent with CAA-ri. Blood was abnormal for PCR (3.11 mg/dl; normal 0–0.5), leucocyte count (37.02 × 10^3^/uL with prevalent neutrophils), creatinine (1.36 mg/dl; normal 0.7–1.2), alkaline phosphatase (193UI/l; normal 40–130), gammaGT (63UI/l; normal 8–61), and LDH (1170UI/l; normal 135–214). EEG revealed diffuse anterior slowing of background activity with superimposed epileptiform paroxysms. Lumbar puncture showed raised proteins (84.3 mg/dl; normal 20–40), IgG (8.7 mg/dl; normal 0–4), albumin (45.6 mg/dl; normal 10–32), and glucose (127.3 mg/dl; normal 40–70) but normal cell count in the CSF. CRP for principal neurotropic agents and onconeural antibodies were negative. Serological tests for principal neurotropic agents and autoimmune encephalitis were negative. He was treated with intravenous dexamethasone (8 mg/d for 15 days followed by a slow dose reduction). One week later, left hemiparesis worsened abruptly. A new brain MRI showed a subacute ischemic insult in right caudate and putamen and internal capsule. Gradually, confusion and hemiparesis improved. Three weeks later, a follow-up brain MRI (Fig. 1o) showed a clear-cut reduction of vasogenic edema and regular evolution of the right ischemic lesion.

## Discussion

CAA-ri is a potentially reversible and treatable encephalopathy [6, 7]. Diagnostic criteria of CAA-ri have been introduced in 2011 [3, 6] and validated in 2016 [6]. Definite diagnosis requires neuropathological evidence of vascular inflammation and amyloid deposition within vessels of the affected area [2]. However, diagnosis of possible or probable CAA-ri may be made using clinical and neuroradiological findings [2, 3, 6, 7, 10], without requiring brain biopsy. Clinical presentation of CAA-ri [6, 7, 9] includes acute/subacute onset of behavioral symptoms, altered level of consciousness, and rapidly progressive cognitive decline, as well as seizures, headache, and focal neurological signs. Furthermore, as described in other studies [11, 12], some CAA-ri patients may be minimally symptomatic or completely asymptomatic. Brain MRI findings include the presence of ≥ 1 of cortical and subcortical hemorrhagic lesions [6, 10, 13] including cerebral macrobleeds, cerebral microbleeds, and/or cortical superficial siderosis, associated with asymmetric unifocal or multifocal corticosubcortical or deep white matter hyperintensities, not depending by previous intracerebral hemorrhage, extending to the immediately subcortical white matter in probable CAA-ri, or with white matter hyperintensity simply extending to the immediately subcortical white matter in possible CAA-ri.

Etiopathogenesis of CAA-ri still remains unknown. Some data support the hypothesis of an inflammatory autoimmune response against cerebrovascular Aβ-amyloid deposits [14]. Arrighi et al. [15] defined the ARIA with vasogenic edema/sulcal effusion (ARIA-E) or hemosiderin deposits (ARIA-H) seen on MRI of patients with Alzheimer’s disease treated with bapineuzumab. These MRI abnormalities share many similarities with the typical MRI findings of CAA-ri [9]. Recent studies [8, 16, 17] identified anti-Aβ autoantibodies in the CSF of CAA-ri patients with high concentrations during the active phase of the disease and subsequent titer reduction after clinical-neuroradiological remission with or without immunotherapy. CSF analysis demonstrates elevated protein in about 71% of patients with CAA-ri and pleocytosis in 45% [2].

Clinical and neuroradiological differential diagnoses of CAA-ri include posterior reversible encephalopathy syndrome, reversible cerebral vasoconstriction syndrome, mitochondrial encephalopathy, lactic acidosis, stroke-like syndrome, Varicella Zoster virus and other infectious vasculitides, sarcoidosis, systemic amyloidosis, giant cell arteritis, primary angiitis of the CNS, and vascular malformations [18, 19]. Patient’s past medical history, clinical presentation, blood and CSF analysis, and neuroradiological findings at brain MRI generally help to achieve a reliable diagnosis.

The MRI features of CAA-ri include bilateral and asymmetrical cortical and subcortical white matter vasogenic edema, associated with diffuse CMBs and/or CSS, mainly involving the temporal and parietal lobes, with a great prevalence of micro-hemorrhages in the area of cerebral vasogenic edema. The proper issue of neuroradiological differential diagnosis of CAA-ri is beyond the scope of this paper and will be addressed elsewhere.

General recommendations on the management of CAA-ri still have to be established. In previous studies, the majority of patients have been treated with high-dose corticosteroids. Auriel et al [6]. provided evidence for using empirical immunosuppressive therapy in patients meeting the criteria for probable CAA-ri and avoiding brain biopsy. Clinical and neuroradiological improvement is reported in most cases after immunosuppression treatment [20–23].

All the patients described herein were older than 40 years and presented with neurological manifestations not directly attributable to an intracerebral hemorrhage. Four patients (patients 1, 2, 4, and 7) had a typical acute presentation including an altered level of consciousness and focal neurological signs. The remaining three patients had an atypical onset, with only mild and transient neurological symptoms in two (patients 3 and 4) or without an acute/subacute clinical presentation (patient 6). While CAA-ri is usually associated with marked neurological symptoms, some of our patients did not, resembling the often paucisymptomatic or asymptomatic patients with ARIA [15]. Brain MRI of all the patients revealed asymmetric unilateral or bilateral T2-weighted or FLAIR subcortical white matter hyperintensities extending to the immediately subcortical white matter associated with hemorrhagic findings typical of cerebral amyloid angiopathy predominantly in the affected areas. However, two patients (patients 1 and 2), one of which (patient 1) before brain MRI, underwent cerebral DSA to rule out vascular malformations and signs of cerebral vasculitis, and in two other patients (patients 5 and 6), PRES was the first neuroradiological hypothesis, in one of which (patient 6) for the confounding history of previous renal transplantation. In the case series presented herein, the main difficulty in recognizing CAA-ri MRI findings and proposing the correct diagnosis seem to be related firstly to the unit of neuroradiology’s knowledge of clinical and MRI presentation of CAA-ri, as well as the related curve of knowledge of the neuroradiologists, and secondly to specific patients' history.

CSF analysis has been performed in only two patients, and the results were compatible with the data reported in the literature. The lack of CSF analysis, as occurred in the remaining five patients reported herein, may represent a further lack of some supportive data that are indeed needed to rule out or confirm the diagnosis of CAA-ri. In daily clinical practice, this may result in confounding interpretation of symptoms and initial misdiagnosis.

Four patients showed significant clinical and neuroradiological improvement after corticosteroid therapy. The remaining three patients experienced a spontaneous remission of neurological symptoms and/or significant reduction of brain edema at MRI. To the best of our knowledge, only a few cases with improvement in the absence of immunotherapy have been described [11, 20].

In conclusion, the patients reported herein highlight several points of interest in CAA-ri. First, a dissociation between mild or absent acute neurological presentation and brain MRI abnormalities is possible. This clinical-neuroradiological dissociation could corroborate prior reports presenting CAA-ri as a human spontaneous model of ARIA [14, 15]. Notably, CAA-ri generally presents with consistent neurological symptoms, whereas ARIA is often asymptomatic or mild [15]. Second, clinical evaluation, brain MRI, and CSF are the keys to the diagnosis of CAA-ri and to undertaking an immunosuppressive therapy, avoiding brain biopsy. Notably, currently brain MRI might be considered the “gold standard” examination for a reliable diagnosis of CAA-ri and for distinguishing it from other neurological conditions. Third, other examinations from CSF analysis and ApoE genotype are helpful to support the diagnosis, however they are not currently included in the diagnostic criteria of CAA-ri. Further studies are needed in order to introduce the possible use of the titer of CSF anti-Aβ antibodies in the CAA-ri diagnosis and monitoring. Fourth, CAA-ri is a typically steroid-responsive brain dysfunction. However, in the population presented herein, three patients with mild neurological symptoms had spontaneous clinical and neuroradiological improvement without immunosuppressive treatment. This seems consistent with the interpretation of CAA-ri as a mechanism of amyloid clearance, such as proposed by other authors [11, 12].
